# PROXIMAL HUMERAL LOCKING PLATE: A VIABLE ALTERNATIVE FOR FIXATION OF DISTAL FEMORAL FRACTURES IN CHILDREN

**DOI:** 10.1590/1413-785220233102e262167

**Published:** 2023-06-09

**Authors:** WEVERLEY RUBELE VALENZA, JAMIL FAISSAL SONI, BRUNO HENRIQUE SCHUTA BODANESE, DEIVIDE MATEUS ROSSETTO, FRANCISCO GUILHERME DE PAULA KOSOVITS, PEDRO IVO PEDRONI CORDEIRO

**Affiliations:** 1. Hospital do Trabalhador de Curitiba, Curitiba, PR, Brazil.; 2. Hospital Universitário Evangélico Mackenzie, Curitiba, PR, Brazil.

**Keywords:** Surgical Procedures, Operative, Femoral Fractures, Children, Procedimentos cirúrgicos operatórios, Fraturas do fêmur Crianças

## Abstract

Distal femoral metaphyseal fractures are rare in children, and the proximity of the fracture to the growth plate makes their approach challenging.

**Objective:**

Evaluate outcomes and complications of treatment of distal femoral metaphyseal fractures in children with proximal humeral locking plates.

**Method:**

Retrospective study between 2018 and 2021, including seven patients. The analysis included general characteristics, trauma mechanism, classification, clinical and radiographic outcomes, and complications.

**Results:**

The mean follow-up was 20 months, the average age was nine years, five patients were boys, and six fractured on the right side. Five fractures were caused by car accidents, one by falling from their own height and one by playing soccer. Five fractures were classified as 33-M/3.2 and two as 33-M/3.1. Three fractures were open, Gustilo IIIA. All seven patients recovered mobility and resumed their pre-trauma activities. All seven healed, and one fracture was reduced to 5 degrees valgus, without any other complications. Six patients had the implant removed and did not present refracture.

**Conclusion:**

Treatment of distal femoral metaphyseal fractures with proximal humeral locking plates is a viable option that offers good results and fewer complications, saving the epiphyseal cartilage. Level of Evidence II; Controlled study without randomization.

## RESUMO

As fraturas metafisárias distais do fêmur são raras em crianças, a proximidade da fratura com a placa de crescimento torna a sua abordagem desafiadora.

**Objetivo:**

Avaliar resultados e complicações do tratamento das fraturas da metáfise distal do fêmur em crianças com placas de úmero proximal.

**Método:**

Estudo retrospectivo entre 2018 e 2021 incluindo sete pacientes. A análise incluiu características gerais, mecanismo do trauma, classificação, resultados clínicos, radiográficos e complicações.

**Resultados:**

A média do acompanhamento foi de 20 meses, a idade média foi de nove anos, cinco pacientes eram meninos e seis fraturas do lado direito. Cinco fraturas por acidentes automobilísticos, uma por queda da própria altura e uma jogando futebol. Cinco fraturas classificadas como 33-M/3,2 e duas como 33-M/3,1. Três fraturas foram expostas, Gustilo IIIA. Todos os sete pacientes recuperaram a mobilidade e retomaram às atividades anteriores ao trauma. Todas as sete fraturas consolidaram, uma fratura foi reduzida com valgo de 5 graus, e não houveram outras complicações. Seis pacientes tiveram o implante removido e não apresentaram refratura.

**Conclusão:**

O tratamento das fraturas da metáfise distal do fêmur com placas de úmero proximal é uma opção viável que oferece bons resultados com poucas complicações, poupando a cartilagem epifisária. Nível de Evidência II; Estudo controlado sem randomização.

## INTRODUCTION

**Figure 1:**
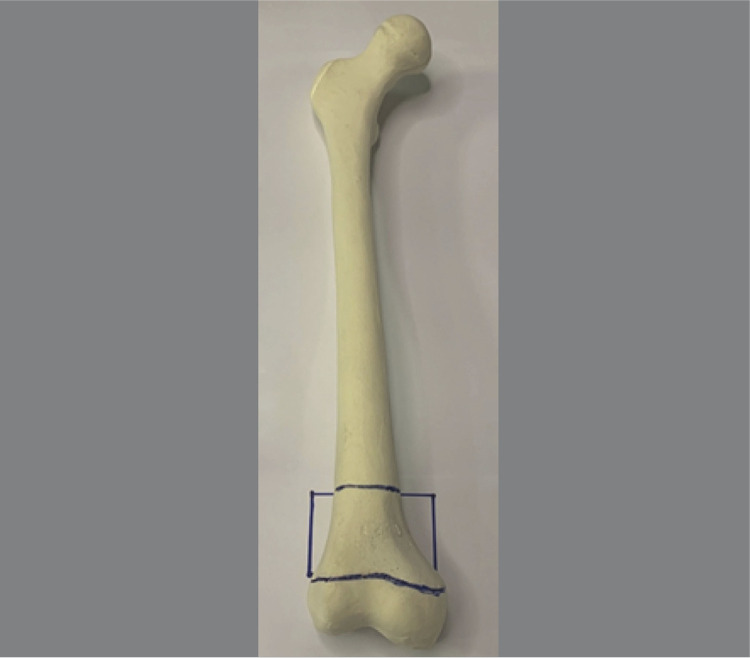
Heim’s square.

Fractures of the distal metaphysis of the femur are relatively uncommon in pediatric patients and correspond to 12% of all femoral fractures in this population.^1^ The treatment choice for these pediatric fractures generally depends on several factors, including age, body weight, trauma energy, and occurrence of associated injuries and open fracture.^2^ Most children younger than 6 years who present with closed fractures and without other injuries should receive conservative treatment with closed reduction and cast immobilization. However, treating these fractures can be challenging in pediatric patients older than 6 years, particularly when surgical fixation is required, since no consensus exists regarding the optimal implantation technique in this setting. The most critical challenges in selecting the implants are to ensure that they offer stability until the fracture consolidates and to prevent physeal injury during insertion of the implant. Often, the area between the fracture line and the distal femoral physis is very narrow, and it becomes difficult to insert the implant without damaging this critical growth region. ( Figure 1 )^3^

The literature describes several techniques for surgical fixation of distal femoral fractures in children. These include percutaneous fixation with Kirschner wires, monolateral and circular external fixation, elastic intramedullary nailing, submuscular bridge plating, and open reduction and internal fixation with plates.^4^

Stabilization of distal femoral fractures using proximal humeral locking plates has been described by Abdelgawad et al.^5^ and is also used by Bor et al.^6^ The shape of the plate adapts well to the lateral cortex of the distal femur, and the design of the plate allows screws to be inserted in multiple planes in this small distal fragment without violating the distal physeal plate.

Based on these considerations, the aim of this study was to evaluate the results and complications of treating distal metaphyseal fractures of the femur using open reduction and fixation with proximal humeral locking plates in children and adolescents.

## MATERIALS AND METHODS

**Figure 2:**
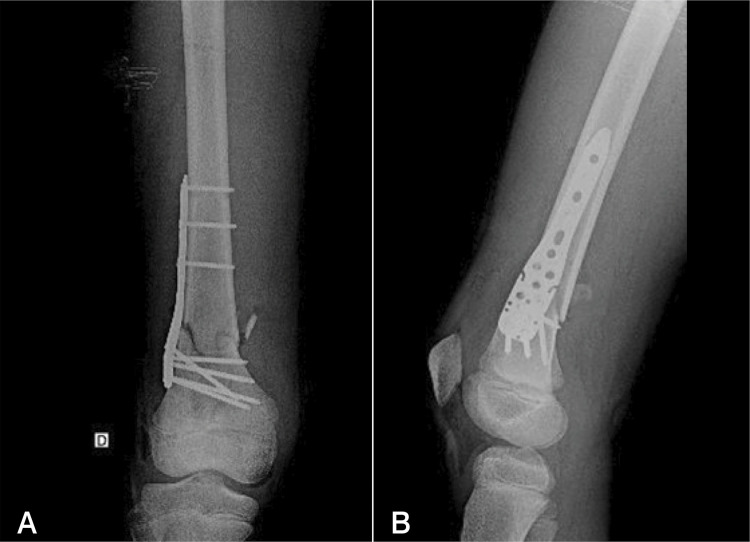
(A and B) Immediate postoperative images of one of the patients with fracture of the distal femoral metaphysis that received fixation with a proximal humeral locking plate. Left: anteroposterior view; right: lateral view.

Retrospective study conducted between 2018 and 2021 at a tertiary trauma hospital. We reviewed the medical records of skeletally immature patients with fractures of the distal end of the femur stabilized with proximal humeral locking plates. ( Figures 2 )

The data analyzed included the patients’ sex and age at the moment of the trauma, the side of the fracture (right or left), the mechanism of the injury, the classification of the fracture according to the AO Pediatric Comprehensive Classification of Long Bone Fractures,^7^ the associated injuries (if present), and the occurrence of open fracture according to Gustilo & Anderson.^8^

We also evaluated whether the patient received any treatment before the definitive fixation, the duration of hospital stay, the occurrence of events during follow-up, and information about removal of the plate.

Outcomes and complications were assessed by the degree of knee joint mobility after surgery, fracture consolidation, return to previous activities, and occurrence of angular deviation, leg length discrepancy, growth disorder, and infection.

We excluded patients with incomplete medical record data, skeletal immaturity, and follow-up shorter than 1 year.

The study’s protocol was approved by the institution’s research ethics committee (CAAE: 41066020.2.0000.5225) according to the Brazilian National Health Council Resolutions 196/96 and 251/97.

## RESULTS

Eight pediatric patients with fractures of the distal end of the femur were treated with open reduction followed by fixation with proximal humeral locking plates between 2018 and 2021 at our institution. After excluding one patient with a follow-up shorter than 1 year, the final analysis included eight patients.

Five of the patients were boys, and six fractures affected the right side. The median age of the patients at the moment of the trauma was 9 years (range 8–13 years). Five patients had fractures due to high-energy trauma from motor vehicle accidents: one was hit by a truck, and three were involved in motorcycle-car, bicycle-car, and car-car collisions. One patient had a spastic cerebral palsy with bone fragility and presented the fracture after falling on the same level, and the another one broke his femur playing soccer, he had a non-ossifying fibroma (NOF).

Three patients had associated injuries - one had an associated ipsilateral leg fracture (floating knee), one presented with associated pulmonary contusion, grade IV liver injury, and iliac wing fracture, and the other had a NOF.

Based on the AO classification, five fractures were classified as 33-M/3.2 and two as 33-M/3.1. Three fractures were open and classified as type IIIA according to Gustilo & Anderson. ( Table 1 )

**Table 1:** General characteristics of the patients included in the study.

Patient	Age (years)	Sex	Mechanism of trauma	AO classification	Type of fracture
1	8	Female	Car-car collision	33-M/3.1	Closed
2	8	Male	Hit by a truck	33-M/3.2	Open GA IIIA
3	9	Female	Fall on the same level	33-M/3.2	Closed
4	12	Male	Bicycle-car collision	33-M/3.2	Open GA IIIA
5	13	Male	Motorcycle-car collision	33-M/3.2	Open GA IIIA
6	10	Male	Playing soccer	33-M/3.1	Closed
7	12	Male	Car-car collision	33-M/3.2	Closed

Source: Authors (2021). Abbreviations: AO classification, AO Pediatric Comprehensive Classification of Long Bone Fractures; GA IIIA, Gustilo & Anderson classification type IIIA.

Five patients had received provisional treatment before the definitive fixation. All three patients with open fractures had undergone cleaning, debridement, and external fixation, while the two other patient - who had a closed femoral fracture due to a car accident - had received external fixation.

The median duration of hospital stay was 9 days (range 2–31 days), and the median duration of follow-up was 20 months (range 12–41 months).

**Figure 3:**
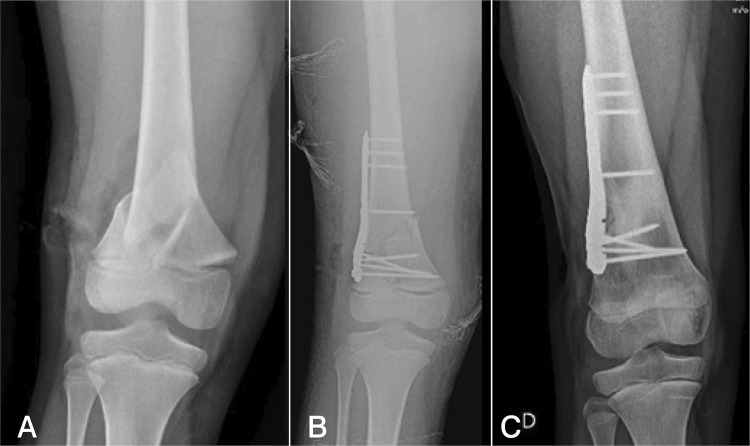
(A, B and C) Preoperative, immediate postoperative, and 1 year after surgical fixation of fracture of the distal end of the femur with posterior humeral locking plating.

All patients present complete recovery of mobility ( Table 2 ), and all fractures consolidated. One patient had a 5-degree valgus deviation that was maintained during fixation and remained until final consolidation, with no deviation in antecurvatum or recurvatum. All seven patients were able to resume the same activity level they had before the fractures. ( Figures 3 )

**Table 2:** Surgical results and complications among the patients included in the study.

Patient	Postoperative mobility	X-ray	Physeal injury	Leg length discrepancy
1	0 – 135^o^	No deviation	No	No
2	0 – 140^o^	No deviation	No	No
3	0 – 130^o^	5-degree valgus	No	No
4	0 – 140^o^	No deviation	No	No
5	0 – 135^o^	No deviation	No	No
6	0 – 140^o^	No deviation	No	No
7	0 – 140^o^	No deviation	No	No

Source: Authors (2021).

There were no cases of infection, physeal injury, or leg length discrepancy.

Six patients had the locking plates removed and presented no refracture. In the remaining two patients, the fractures have consolidated and the removal of the implant has been planned.

## DISCUSSION

Fractures of the distal end of the femur are the most uncommon types of fractures affecting this bone. These fractures usually result from high-energy trauma and develop intrinsic instability when presenting with deviations.^3^ This poses a problem for the surgeon, who must reduce the fracture and offer optimal stabilization until consolidation.

In younger children, fixation of these fractures with crossed Kirschner wires is sufficient. However, older children require a more stable fixation. The narrow fragment between the fracture and the growth plate makes it extremely difficult to obtain stability of the fracture without damaging this critical structure.

Butcher & Hoffman^9^ described 10 fractures of the distal femur treated with reduction and percutaneous fixation with crossed Kirschner wires. Six patients presented good results, two progressed with a flexion deformity of 10 degrees, one progressed with a valgus deformity of 6 degrees, and one patient had a transient common peroneal nerve palsy. Although crossed-wire fixation is an excellent method to treat younger children with distal femoral fractures, this therapeutic strategy is not sufficient to stabilize the fixation in adolescents.

Canavese et al.^3^ treated 24 patients with fractures of the distal femoral metaphysis using elastic stable intramedullary nailing and observed two pseudoarthrosis and three fracture deviations. Long leg casting was used for 3 to 4 weeks to reduce pain and prevent further deviations.

Li et al.^10^ compared the treatment of distal femoral fractures using external fixation versus elastic stable intramedullary nailing and observed similar clinical and radiographic results with both techniques. Compared with elastic intramedullary nailing, external fixation had the advantage of a shorter operative time and obviated the need for another surgical procedure for hardware removal, but had the disadvantages of more frequent pin tract infection, soft tissue irritation, and pain site scarring. External fixation was also associated with two refractures within 1 month from the hardware removal.

Sabharwal^11^ reported optimal results after treating five fractures of the distal femoral metaphysis using circular external fixation. The author reported no need for pin repositioning due to infection, and all patients recovered knee and hip mobility 3 months after the fixator was removed, while one patient developed transient foot drop.

Kanlic et al.^12^ used submuscular bridge plating to treat 51 femoral fractures, of which only 6% affected the distal metaphysis. The authors reported good results with the method. However, they described one patient in whom only one plate screw (PCL) would be available for fixation of the distal fragment and proceeded with fixation of the epiphysis using two screws to ensure more stability but increased the risk of physeal injury. In most distal metaphyseal fractures of the femur in children, fixation of the distal fragment using three screws is not feasible, which is the major problem with conventional compression plates (DCP and LCP).

To increase the stability of the fixation in distal femoral fractures, Lin et al.^4^ described good results and no implant fractures with an interchangeable titanium plate with three screw holes in the distal femoral epiphysis and another plate that adapts to the first one, thus allowing the fixation of both to the proximal fragment of the femur. However, this adaptation of one plate over the other weakens the fixation. Additionally, the fixation of the epiphysis increases the chance of physeal injury.

In an attempt to provide more stability to the fixation of the distal metaphysis fragment without violating the physeal plate, Abdelgawad et al.^5^ described two distal femoral fractures treated with proximal humeral locking plates, reporting good results and no physeal injury.

Proximal humeral locking plates have the advantage of adapting well to the contour of the distal femur, allowing the fixation of the distal fragment with up to six screws, depending on the fracture line. The screws can be placed in multiple planes — divergent, convergent, and straight — providing greater stability. A disadvantage is the width of 3.5 mm of the humeral plate, which may not offer sufficient stability in larger patients.

In our series, five fractures were comminuted and two was transverse. Five patients had suffered high-energy trauma. After the initial stabilization, all fractures consolidated without further deviation. Fixation of a fracture in a patient with cerebral palsy and bone fragility was performed with a residual valgus deformity of 5 degrees, which was maintained until final consolidation. We observed no physeal injuries, angular deviations, or leg length discrepancies.

Three fractures were open, and none of the patients presented postoperative infection or vascular or nerve damage. All patients returned to the activity levels that they had before the trauma.

**Figure 4:**
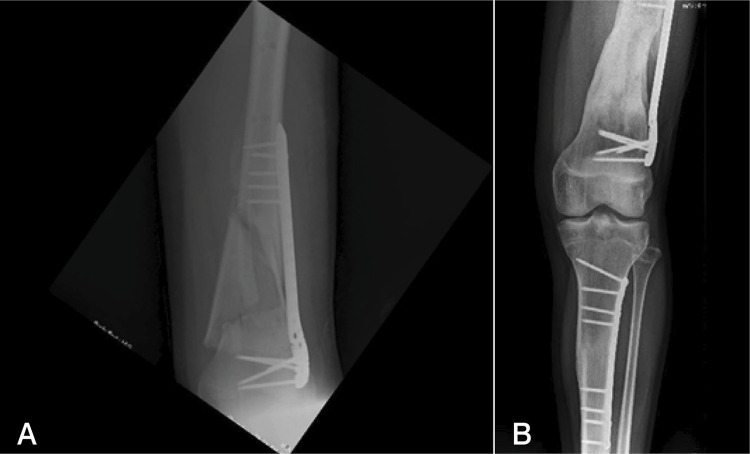
(A and B) A 13-year-old patient with floating knee and an open comminuted femoral fracture (grade IIIA). Left, immediate postoperative period; right, 13 months after surgery.

A 13-year-old patient with floating knee had an open comminuted grade IIIA fracture. Even though the proximal humeral plate was only 3.5 mm wide, it provided sufficient stability for consolidation of the fracture without further deviation in this patient. ( Figures 4 )

We removed the plates from six patients, while the remaining one patient is waiting to schedule the procedure to avoid valgus deviation due to plate retention, as described by Kelly et al.^13^

The present study is limited by the retrospective design and the small number of cases, explained by the rarity of these fractures. Compared with other studies in the literature evaluating proximal humeral locking plates to treat distal femoral fractures in children, ours is the one with the largest number of patients.

## CONCLUSION

In conclusion, proximal humeral locking plating is a viable fixation option to treat distal femoral metaphyseal fractures in children and adolescents without violating the physis and yielding good results with few complications.
